# Preclinical modeling of low energy X-rays radiological burn: Dosimetry study by monte carlo simulations and EPR spectroscopy

**DOI:** 10.3389/fphys.2022.1075665

**Published:** 2022-12-08

**Authors:** Manon Guillou, Bruno L’Homme, François Trompier, Gaëtan Gruel, Yolanda Prezado, Morgane Dos Santos

**Affiliations:** ^1^ Institut de Radioprotection et de Sureté Nucléaire (IRSN), PSE-SANTE/SERAMED/LRAcc, Fontenay-aux-Roses, France; ^2^ Institut de Radioprotection et de Sureté Nucléaire (IRSN), PSE-SANTE/SDOS/LDRI, Fontenay-aux-Roses, France; ^3^ Institut Curie, University Paris Saclay, PSL Research University, Inserm U 1021-CNRS UMR 3347, Orsay, France

**Keywords:** dosimetry, low energy X-rays, interventional radiology, radiological burn, preclinical model, EPR spectroscopy, Monte Carlo simulation

## Abstract

Interventional radiology has grown considerably over the last decades and become an essential tool for treatment or diagnosis. This technique is mostly beneficial and mastered but accidental overexposure can occur and lead to the appearance of deterministic effects. The lack of knowledge about the radiobiological consequences for the low-energy X-rays used for these practices makes the prognosis very uncertain for the different tissues. In order to improve the radiation protection of patients and better predict the risk of complications, we implemented a new preclinical mouse model to mimic radiological burn in interventional radiology and performed a complete characterization of the dose deposition. A new setup and collimator were designed to irradiate the hind legs of 15 mice at 30 Gy in air kerma at 80 kV. After irradiation, mice tibias were collected to evaluate bone dose by Electron Paramagnetic Resonance (EPR) spectroscopy measurements. Monte Carlo simulations with Geant4 were performed in simplified and voxelized phantoms to characterize the dose deposition in different tissues and evaluate the characteristics of secondary electrons (energy, path, momentum). 30 mice tibias were collected for EPR analysis. An average absorbed dose of 194.0 ± 27.0 Gy was measured in bone initially irradiated at 30 Gy in air kerma. A bone to air conversion factor of 6.5 ± 0.9 was determined. Inter sample and inter mice variability has been estimated to 13.9%. Monte Carlo simulations shown the heterogeneity of the dose deposition for these low X-rays energies and the dose enhancement in dense tissue. The specificities of the secondary electrons were studied and showed the influence of the tissue density on energies and paths. A good agreement between the experimental and calculated bone to air conversion factor was obtained. A new preclinical model allowing to perform radiological burn in interventional radiology-like conditions was implemented. For the development of new preclinical radiobiological model where the exact knowledge of the dose deposited in the different tissues is essential, the complementarity of Monte Carlo simulations and experimental measurements for the dosimetric characterization has proven to be a considerable asset.

## 1 Introduction

In the last 60 years, interventional radiology has become an essential tool for treatment (heart disease, neuroradiology, *etc.*) or for diagnosis, with a significant diversification of the type of procedures performed, thus considerably increasing the number of procedures performed (Midulla et al., 2019). Interventional radiology includes a large range of procedures under imaging guidance or control using low-energy X-rays, from 70 to 120 kV. These procedures represent an undeniable asset for patients (less invasive, reduction of hospitalization time, *etc.*), and are mostly beneficial and mastered. Nevertheless, due to the complexity of the act, the repetition of exposure, the necessity to treat the patients and also the lack of standardization of practices, some procedures can be very long and lead to radiation overexposures which most often result in local skin injuries (International Commission on Radiological Protection, 2000; Coeytaux et al., 2015; Jaschke et al., 2017). Indeed, when these exposures exceed a certain dose threshold (>10 Gy), deterministic effects can appear. The most common effects are observed in the skin and superficial soft tissues, such as dry or wet erythema, alopecia or sometimes tissue necrosis (International Atomic Energy Agency, 1998). However, as photoelectric effect is the dominant physical interaction of low-energy X-rays (<100 keV) and as dose deposition depends also on the composition and density of the tissues traversed (because proportional in low energy range to Z^4^, whereas varying in Z and Z^2^ respectively for Compton and pair production), the doses deposited to the bone and closely related tissues are expected to be higher up to a factor of 9, because of higher density with higher Z materials such as calcium. Therefore, even if the dose on soft tissues may be lower than the dose threshold for soft tissue necrosis (25 Gy (International Atomic Energy Agency, 1998)), the threshold dose for bone necrosis (40 Gy) may be approached or exceeded and can lead to severe complications (Haute Autorité de Santé, 2014). Although bone is usually not immediately identified as a tissue at risk in the case of accidental overexposures in interventional radiology, as most of the time the doses involved remain below the threshold of severe deterministic effect in soft tissue, at longer term, it has already been shown that high doses delivered to the bone during radiotherapy protocols (Ugurluer et al., 2014; Frankart et al., 2021) or radiological accident with sources (Clairand et al., 2008) can lead to severe clinical consequences such as fractures or osteoradionecrosis, considerably altering the quality of life of patients. The risks associated with high bone dose exposures should therefore not be neglected, whatever the medical exposure considered.

Radiation protection of patients in the field of interventional radiology has therefore become a major concern and better knowledge about the biological consequences of this type of exposure on different types of tissue (skin, muscle and bone) is required. The development of a preclinical experimental model is therefore essential.

To conduct these radiobiological studies, it is possible to use conventional X-ray cabinets or small animal irradiators which have been used more widely in the last 2 decades (Brown et al., 2022). These platforms are mainly developed to mimic radiation therapy protocols and allow delivery of very small irradiation fields (mm) with very small penumbra and have the advantage of being more attenuated as they involve medium to low-energy X-rays (kV). However, several studies carried out by Monte Carlo (MC) simulations have already shown the specificities on the dose distribution and the dose enhancement on bone for X-rays over 100 kV (11–13). This phenomenon is even more significant with the reduction of the kilovoltage of photon beams below 100 kV. Thus, in this energy range, specific MC simulations are required to evaluate the dose deposition and the dosimetric characteristics (mean dose, heterogeneities, secondary electrons) and additional experimental dosimetry measurements are a considerable asset to know the exact delivery dose on dense tissues. In particular, it is possible to estimate the actual bone dose thanks to the Electron Paramagnetic Resonance (EPR) spectroscopy. EPR is a powerful analytical technique based on the detection of paramagnetic species (like free radicals) that can be radio-induced in solid materials and also in biological tissues like enamel, nails, hairs or bone (Brady et al., 1968; Trompier et al., 2009; Krefft et al., 2014). This technique is used for retrospective dosimetry for dose reconstruction and has been used successfully on bone tissue in case of accidental exposure to ionizing radiation to estimate the radiation dose (International Atomic Energy Agency, 2004; Clairand et al., 2006; Trompier et al., 2007).

The aim of this work was to develop and characterize a new preclinical mouse model mimicking radiological burn in interventional radiology to carry out radiobiological studies and to study the radiopathological specificities of this type of exposure at low energy. A specific setup was developed on the SARRP (Small Animal Radiation Research Platform, XSTRAHL, Ldt. (Wong et al., 2008)) at 80 kV with the design of a new collimator allowing local hind leg irradiation on mice. The whole installation was modeled with the Geant4 MC code to characterize the dose deposition in the different tissues and study the specificities of the dose deposition, the secondary electron spectrum and the electron paths. In addition, experimental bone dose assessments were performed by EPR spectroscopy to study its variability and investigate the correlation with bone density/composition.

## 2 Material and methods

### 2.1 Irradiation platform and experimental dosimetry

To mimic interventional radiology conditions, the Small Animal Radiation Research Platform (SARRP, XSTRAHL Ltd, United Kingdom) was used (Wong et al., 2008). The X-rays source is a conventional Varian X-ray tube (NDI-225–22, NDI, Washington, DC) with an inherent filtration of 0.8 mm of beryllium, a large focal spot size of 3 mm and a beam divergence of 20°. Irradiations were performed at 80 kV, 24 mA with an additional filtration of 0.15 mm of copper to mimic the energy spectrum used in interventional radiology. The Half Value Layer (HVL) is 0.138 ± 0.006 mm of copper, measured by following the protocol described in Dos Santos et al. (Dos Santos et al., 2021a) adapted from the AAPM TG61 protocol (Ma et al., 2001).

**FIGURE 1:**
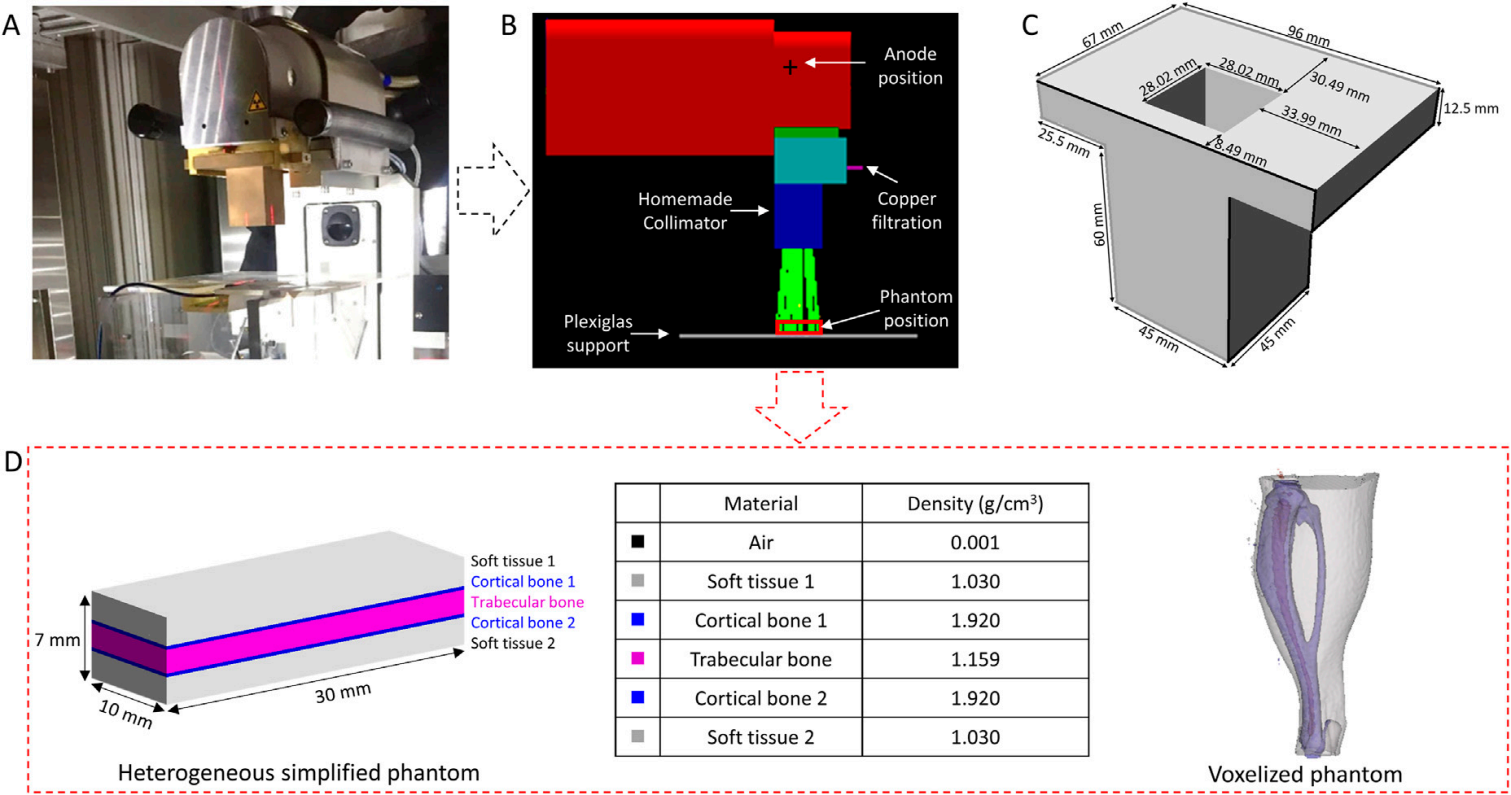
Irradiation setup **(A)**, Geometry implemented on Geant4 **(B)**, homemade brass collimator **(C)** and simplified heterogeneous phantom and voxelized phantom introduced on the Geant4 **(D)**.

For mouse irradiations, a homemade brass collimator was designed by means of MC simulations (Figure 1C) providing an irradiation field of 4.3 × 4.3 cm at 22.5 cm from the source. Reference dosimetry measurements were performed using a PTW TM23342 ionization chamber calibrated in term of air kerma (K_air_). A mean dose rate of 1.66 ± 0.07 Gy min^−1^ was determined (Figure 1A).

EBT3 radiochromic films were used to characterize dose profiles resulting from the irradiations with the designed collimator, as well as to validate the geometry implemented on the MC calculations. The protocol used for EBT3 film analysis is described in Dos Santos et al. (Dos Santos et al., 2021a). Briefly, films were cut at least 48 h before irradiation and scanned at least 24 h after irradiation in a V700 Epson scanner in transmission mode at 150 dpi. A calibration curve between 0 and 3 Gy in K_air_ was constructed and fitted with a fourth-degree polynomial curve.

### 2.2 *In vivo* experiments

*Ethical statement:* adult male C57bl6 mice from Janvier Labs (Le Genest-Saint Isle, France) were used for experiments. Animals were housed at the IRSN animal facilities accredited by the French Ministry of Agriculture for performing experiments on rodents. Animal experiments were performed in compliance with French and European regulations on protection of animals used for scientific purposes (EC Directive 2010/63/EU and French Decree 2013–118) and in compliance with the guidelines and regulations of ARRIVE guidelines. All experiments were approved by the Ethics Committee #81 (approval number E92-032–01) and authorized by the French Ministry of Research under the reference APAFIS#16160–2018071810588014 v2.

*Irradiation and bone conditioning:* localized irradiation of the posterior paw (left and right) of mice were performed with a dose of 30 Gy in K_air_. During irradiation, mice were anesthetized with 100 mg/kg ketamine (Imalgene 1,000, Merial, Lyon, France) and 10 mg/kg xylazine (Rompun^®^ 2%, Bayer Healthcare, Loos, France). Just after irradiation, animals were sacrificed by cervical dislocation (still anesthetized), tibias were harvested, cleaned, flushed (marrow), dried and cut into small pieces for dosimetry measurements in order to experimentally determine the absorbed bone dose.

### 2.3 Electron paramagnetic resonance (EPR) measurements and bone dose estimation

Bone dose assessment was performed using Electron Paramagnetic Resonance (EPR) spectroscopy with an X-band spectrometer (MS5000, Magnettech). Several publications have already described, in detail, the EPR spectroscopy and associated dosimetry principles (Abragam and Bleaney, 1970; International Atomic Energy Agency, 2002; Trompier et al., 2009). Briefly, this method is based on the proportional relationship between concentration of stable radio-induced free radicals and the dose absorbed in the irradiated material. Indeed, when a material is irradiated, there is a creation of free radicals and in the case of calcified tissues such as bone, mainly composed of hydroxyapatite, irradiations create different long-lived stable free radicals (mostly CO_2_^−^), from ionization of impurities of CO_3_^2-^ (International Atomic Energy Agency, 2002; Trompier et al., 2009).

In this work, EPR spectra were recorded at room temperature with a magnetic field between 334 and 337 mT, a modulation of 0.5 or 0.2 mT, a power of 14 dB, five accumulations of 60 s and three to five measurements per bone for each sample.

For bone dose estimation, the so called additive dose method was used (International Atomic Energy Agency, 2002). This method consists in re-irradiating bone samples with known doses to derive by linear regression the initial bone dose estimation. The peak to peak amplitude between the first and third extrema of the EPR spectra is directly proportional to the concentration of free radicals and therefore to absorbed dose. Although this approach is time consuming, we can produce a calibration curve for the sample itself and evaluate the bone dose sensitivity for each sample and therefore the overall variability between samples.

Post-irradiations were performed with a 4 MV X-rays medical linear accelerator (Elekta Synergy^®^). All irradiations were performed in air under electronic equilibrium conditions, given that at this energy air kerma is almost equal to bone kerma providing therefore a dose estimation in terms of bone kerma.

### 2.4 Monte carlo simulations

Calculations were performed with the Geant4 MC code (version G4.10.06. p01) which is an open-source library coded in C++, specialized in the transport of particles (Agostinelli et al., 2003; Allison et al., 2006). It has a large selection of physical processes (electromagnetic, optical interactions, *etc.*) and a large energy range (from several eV to several TeV). For these calculations, the Livermore Electromagnetic physics list was used, an energy cut of 250 eV was considered for all particles. The geometry of the SARRP was implemented on the simulation from the manufacturer’s plans (Figure 1B) to model the irradiation source, create a virtual source, design the collimator, and evaluate the dose deposition in the mouse leg.

*Virtual source model:* To save computing time, a virtual source was modeled and implemented. MC simulations were split into two parts: the first one consists in the simulation of the interaction of 80 keV electrons on the tungsten anode where information about the positions, momentums and the energy of photons was collected (PSF 1) and the second one consists in the simulation of these resulting photons through the two filtrations (0.8 mm Be + 0.15 mm Cu) where information about the positions, momentums and the energy of photons was also collected (PSF 2). From the information of these two-phase spaces, a virtual source was modeled and placed at the anode position allowing to substitute all the elements of the producing the beam (electron beam, tungsten anode and filtrations). This virtual source was studied by comparing: i) the resulting energy spectrum to the one given by the SpekCalc software (Poludniowski et al., 2009), ii) the simulated half value layer (HVL) to the experimental and the SpekCalc HVL and iii) the photon momentums between real and virtual source.

*HVL estimation:* To estimate the HVL by Monte Carlo simulation, the same collimator as the one used for the experimental HVL measurements was implemented (1 × 1 cm^2^ irradiation field) and plates with different thicknesses of copper (no attenuator, 0.05, 0.1, 0.12, 0.142, 0.2, 0.22, 0.24 and 0.3 mm) were introduced in the calculations. The K_air_ at ionization chamber position was calculated using an air cube of 0.9 mm × 0.9 mm x 0.9 mm = 0.729 mm^3^. For each point, 48 billion of histories were simulated leading to a statistical uncertainty less than 0.4%. The Monte Carlo results were fitted with a quadratic fit to estimate the simulated HVL.

*Dose profile characterization:* In order to have an irradiation field of 4 cm × 4 cm at 22.5 cm, simulations were performed in a voxelized phantom of 1 mm × 70 mm x 70 mm composed of 1 mm^3^ water voxels placed on the Plexiglas support (SSD = 22.5 cm). The resulting dose profiles were then compared to the experimental dose profiles obtained with EBT3 radiochromic films.

*Dose computation:* To save computing time and in order to gather significant information about the dose deposition as well as the energy spectrum, momentum and path of the secondary electrons in the different tissues of the mouse leg, simplified rectangular phantoms were introduced in the simulation. These phantoms are composed of 70 slices of 0.1 mm × 30 mm x 10 mm (thickness of 7 mm, volume of 2.1 cm^3^). Two types of simplified phantoms were simulated: i) an homogeneous phantom composed of air, soft tissue, cortical bone or trabecular bone or ii) an heterogeneous phantom, more representative of the mouse leg, composed of 23 slices of soft tissue, three slices of cortical bone, 19 slices of trabecular bone, three slices of cortical bone and 22 slices of soft tissues as illustrated in Figure 1D. The thickness for each tissue (soft tissue, cortical ad trabecular bone) was determined from measurements made on a microCT image at the tibial head. For dose computation, a large number of histories (4 billion) were simulated in order to have a relative dose error less than 1%.

Then, the dose deposition was also calculated in a voxelized mouse leg phantom (Figure 1D). CT images were acquired on the Quantum GX2 (Perkin Elmer) with voxel size of 90 μm, exported in DICOM format, segmented with Slicer 5.0.2 software (Fedorov et al., 2012) to distinguish soft tissue, bone and marrow, and introduced in the simulation.

## 3 Results

### 3.1 Experimental bone dose estimation

Table 1 reports the bone dose estimation based on EPR signal measurements on mouse tibia collected the day of irradiation and exposed to a single dose of 30 Gy in terms of K_air_:

**TABLE 1:** Bone dose estimation by EPR spectroscopy the day of irradiation.

Mouse number	Right tibia bone dose (Gy)	Left tibia bone dose (Gy)
Mouse_01	189.2 ± 10.2	228.4 ± 20.5
Mouse_02	179.6 ± 6.7	184.8 ± 12.5
Mouse_03	171.1 ± 17.5	224.8 ± 19.7
Mouse_04	166.5 ± 10.2	186.6 ± 10.3
Mouse_05	262.5 ± 26.8	247.8 ± 15.1
Mouse_06	156.3 ± 15.0	183.9 ± 7.8
Mouse_07	163.5 ± 7.6	223.6 ± 9.5
Mouse_08	182.1 ± 11.0	162.9 ± 11.1
Mouse_09	189.2 ± 8.8	188.2 ± 8.2
Mouse_10	226.7 ± 16.8	221.5 ± 18.7
Mouse_11	183.4 ± 13.9	198.0 ± 17.1
Mouse_12	182.9 ± 8.8	218.7 ± 4.9
Mouse_13	164.6 ± 7.4	166.0 ± 9.7
Mouse_14	182.0 ± 17.1	Broken
Mouse_15	198.1 ± 9.8	193.9 ± 13.5

An average absorbed dose of 186.5 ± 27.0 Gy and 202.1 ± 25.4 Gy was found respectively in right and left tibia bones initially exposed to 30 Gy (K_air_) with 80 kV X-rays on the SARRP. The relative uncertainties for bone dose estimation for each mousse range between 2.2% and 10.2% and is mainly depending on the uncertainty of the parameters of the fit by linear regression on the dose additive curve. The absorbed dose in bone being the ratio between the intercept and the slope of the fit.

From these data, we can therefore calculate a bone to air kerma conversion factor which is of 6.2 ± 0.9 and 6.7 ± 0.9 respectively for right and left tibia bones, respectively. The coefficient of variations for each group are about 14.5% and 12.6% for right and left tibia bones respectively, highlighting the inter-mouse variability.

These results also allow us to evaluate the intra-mouse variability, as left and right tibia bones were exposed at the same time. The relative intra-mouse variability range between 0.4% and 21.9% with a mean intra-mouse relative variability of 8%. Thus, the intra- and inter-mouse variabilities are of the same order of magnitude.

Combining all the data, a mean absorbed dose in bone of 194.0 ± 27.0 Gy and an averaged conversion factor of 6.5 ± 0.9 between the dose determined in air kerma free in air using an ionization chamber (reference dosimetry) and the dose deposited in mouse bone were determined (Coefficient of variation = 13.9%).

### 3.2 Virtual source model definition

**FIGURE 2:**
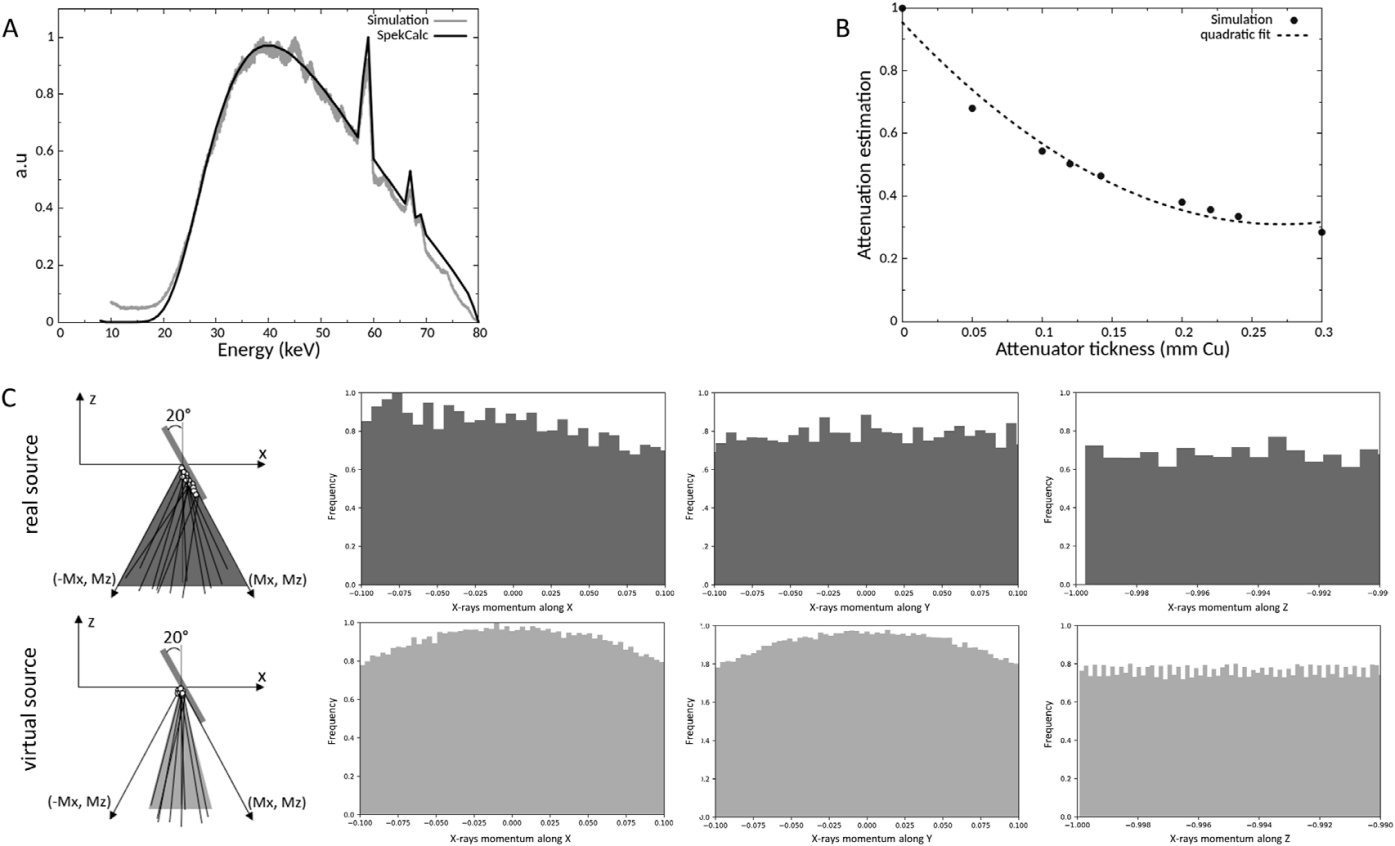
Comparison of the simulated and a theorical energy spectrum at 80 kV **(A)**, determination of simulated half value layer **(B)** and representation of the X-rays momentums of the real and virtual source **(C)**.

Using the data extracted from the two PSF, the source was characterized, and a source model was created as reported in Figure 2. First, we compared the simulated energy spectrum to a theoretical energy spectrum obtained with the SpekCalc software (Poludniowski et al., 2009). There was good agreement between the two energy spectra with an average relative difference less than 5% between 23 and 70 keV, the difference is more important outside this range but only concerns a small proportion of X-rays (Figure 2A). In addition, the half value layer was modeled and estimated at 0.124 ± 0.001 mm of copper (Figure 2B). Experimentally on the SARRP, the measured HVL was about 0.138 ± 0.006 mm of copper and the SpekCalc software estimates an HVL of 0.142 mm of copper. Good agreement was found between all the data with a relative difference of 7.0%. The momentums of the photons were also analyzed to create a source model represented in Figure 2C. To determine the beam divergence of the virtual source, the positions and momentums of the X-rays were studied after the different collimations and, at the phantom position level, the x and y momentums are between -0.1 and 0.1 and for z between -1 and -0.99. From this information and in order to save computing time, we have chosen to reduce the divergence of the source and, following the *X* direction, the anode inclination was not taken into account. Finally, a Gaussian source with a full width at half maximum (FWHM) of 2.3 mm, divergence of 10°, and located at the anode position has been implemented. The analysis of the simulated dose profile described in the next section will allow to evaluate the impact of these modifications.

### 3.3 Dose profile characterization

**FIGURE 3:**
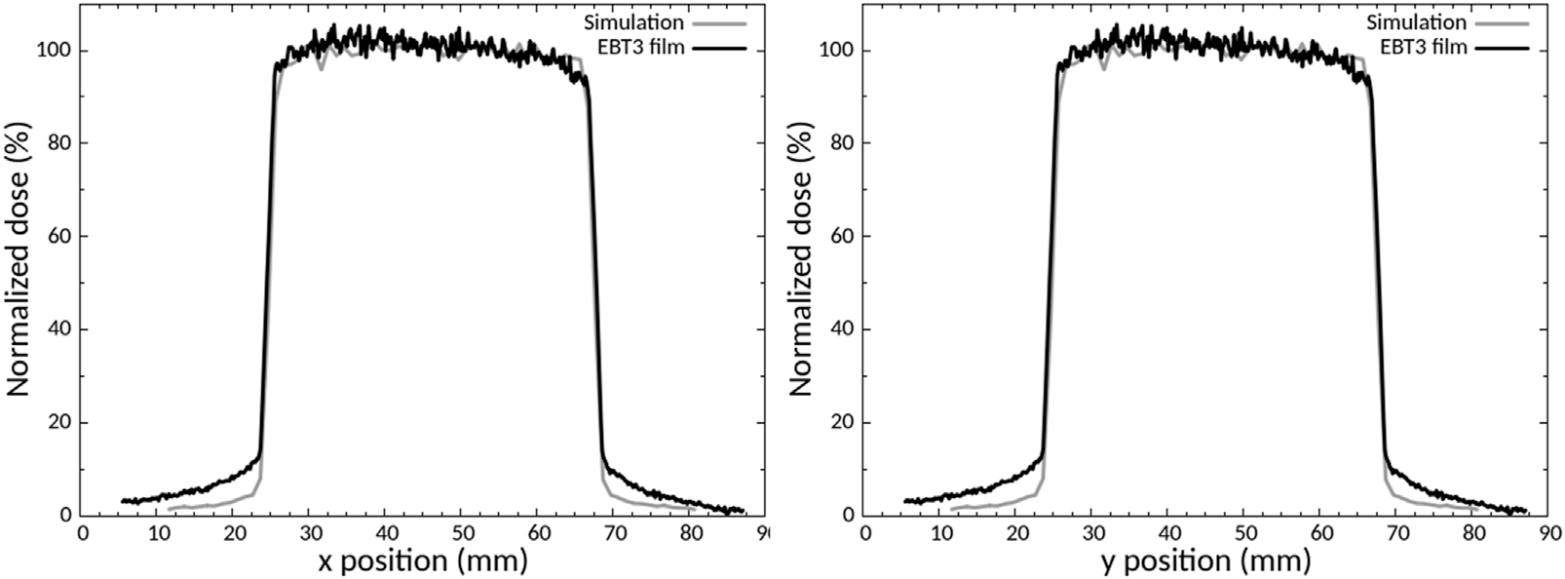
Comparison between experimental and calculated dose profiles along the *x* and *y* axes.

A specific collimator in brass was designed by Monte Carlo simulations (Figure 1C), manufactured, and then characterized experimentally with EBT3 radiochromic films. Figure 3 shows the comparison between experimental and simulated dose profiles along the *x* and *y* axes extracted in the center of the irradiation field and Table 2 reports the data for the evaluation of the FWHM and beam fringe parameters.

**TABLE 2:** full width at half maximum (FWHM) and beam fringe evaluation.

	X profile			Y profile		
	EBT3 film	Simulation	Deviation	EBT3 film	Simulation	Deviation
FWHM (mm)	43.28	42.68	0.605 (1.40%)	42.95	42.67	0.277 (0.65%)
Beam fringe left (mm)	1.01	1.06	0.051	0.92	0.97	0.045
Beam fringe right (mm)	0.81	0.87	0.062	0.81	0.87	0.090

The comparison between the dose profiles is based on the criteria defined by the International Atomic Energy Agency (International Atomic Energy Agency, 2004) (IAEA). There was good agreement between the experimental and simulated dose profile, for the FWHM and beam fringe (Table 3), the build-up region (<2%) and the maximal dose outside the central beam axis (<2%), in compliance with IAEA criteria. The main difference between the profiles is the outside beam edge criterion of up to 50% (30% recommended by IAEA).

**TABLE 3:** Mean normalized dose, attenuation, and tissue to air conversion factor in the simplified heterogeneous phantom.

Tissues	Thickness (mm)	Mean normalized dose (%)	Attenuation in the considered tissue (%)	Tissue to air conversion factor
Soft tissue 1	2.30	16.80 ± 0.51	0.70	1.13 ± 0.03
Cortical bone 1	0.30	99.85 ± 0.17	0.10	6.72 ± 0.01
Trabecular bone	1.90	88.76 ± 5.19	17.30	6.00 ± 0.35
Cortical bone 2	0.30	76.88 ± 2.74	6.90	5.17 ± 0.03
Soft tissue 2	2.20	11.52 ± 0.76	25.60	0.77 ± 0.05

### 3.4 Dose distribution in simplified and voxelized phantom

**FIGURE 4:**
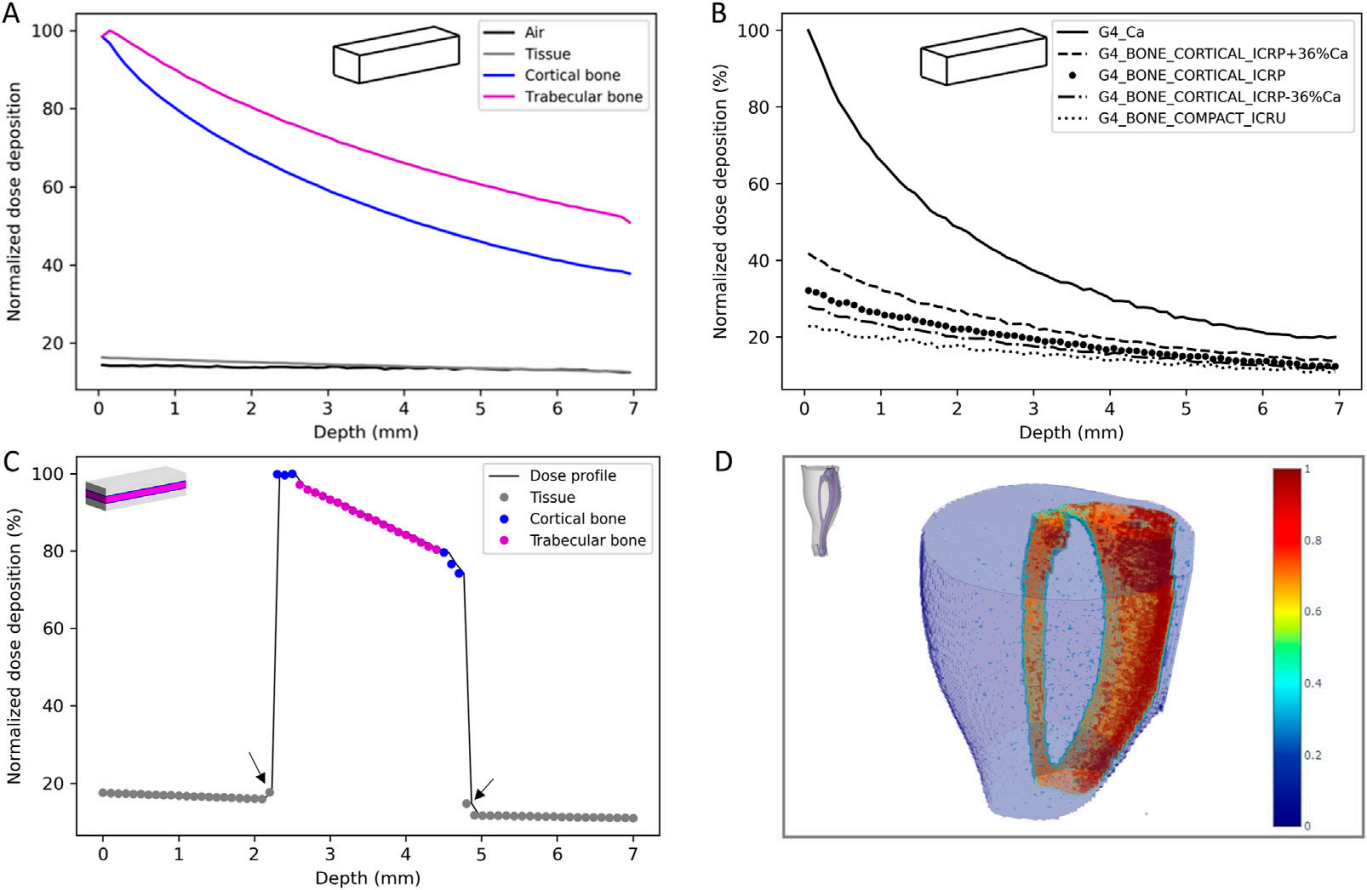
Dose deposition in the homogeneous simplified phantom **(A)** and influence of the bone composition and densit **(B)**, lateral dose profile in the heterogeneous simplified phantom **(C)** and 3D reconstruction of the dose deposition in the voxelized phantom **(D)**.

First, simulations were performed in homogeneous phantoms to evaluate the attenuation of the dose as a function of the density of the material. Attenuation was found to be 13.2, 23.1, 61.6 and 48.3% respectively for air, soft tissue, cortical bone and trabecular bone, for a thickness of 7 mm (Figure 4A). As the attenuation of the dose is different in function of the bone type, to evaluate the impact of the bone composition and density, simplified calculations with different type of bones available in Geant4 and by varying the calcium content were performed (Figure 4B). These results show that the dose deposition and the attenuation depend on the bone density and composition. For bones, the attenuation can vary from 10% to 28% and the dose deposition can vary between 3% and 20% depending on the depth.

Then, simulations on the heterogeneous phantom, more representative of the mouse’s leg, were performed. Table 3 reports the mean normalized dose, the attenuation for each tissue and the tissue to air conversion factor and Figure 4C reports the lateral dose profile obtained in this phantom where grey, blue and pink dots highlight the material considered (soft tissue, cortical bone and trabecular bone respectively).

These results highlight the influence of the density of the materials on the dose deposition and the strong heterogeneity of the dose deposition for these low-energy X-rays. From these data, a bone (trabecular and cortical) to air conversion factor of 6.0 ± 0.5 was calculated. We can also observe a slight increase of the dose deposition in the thin layer of soft tissue in contact with the bone (Figure 4C, black arrows).

Finally, the voxelized phantom, constructed from a mouse leg in our study, provides a 3D reconstruction of the dose deposition and allows more precise evaluation of the bone to air conversion factor in order to compare it to experimental data (Figure 4D). This 3D dose reconstruction also highlights the heterogeneity of the dose deposition and the attenuation in the tissue, especially for bone. The bone, tissue or marrow to air conversion factor were respectively estimated at 5.9, 1.0 and 1.6 with a coefficient of variation of 23%, 29% and 29%. One can note that the dose to the marrow is higher than the dose to the tissue, showing that the secondary electrons created in the bone contribute also to the dose deposition in the marrow. Concerning the bone, there was good agreement between simulated and experimental data (6.0 ± 0.5 *versus* 6.5 ± 0.9). Moreover, if we consider only the cortical part of the tibia bone, i.e., excluding the tibial head, as for experimental measurement the marrow is flushed, a bone to air conversion factor of 6.2 was achieved (CV = 16%) that is in better agreement with experimental data.

### 3.5 Secondary electron characterization

**FIGURE 5:**
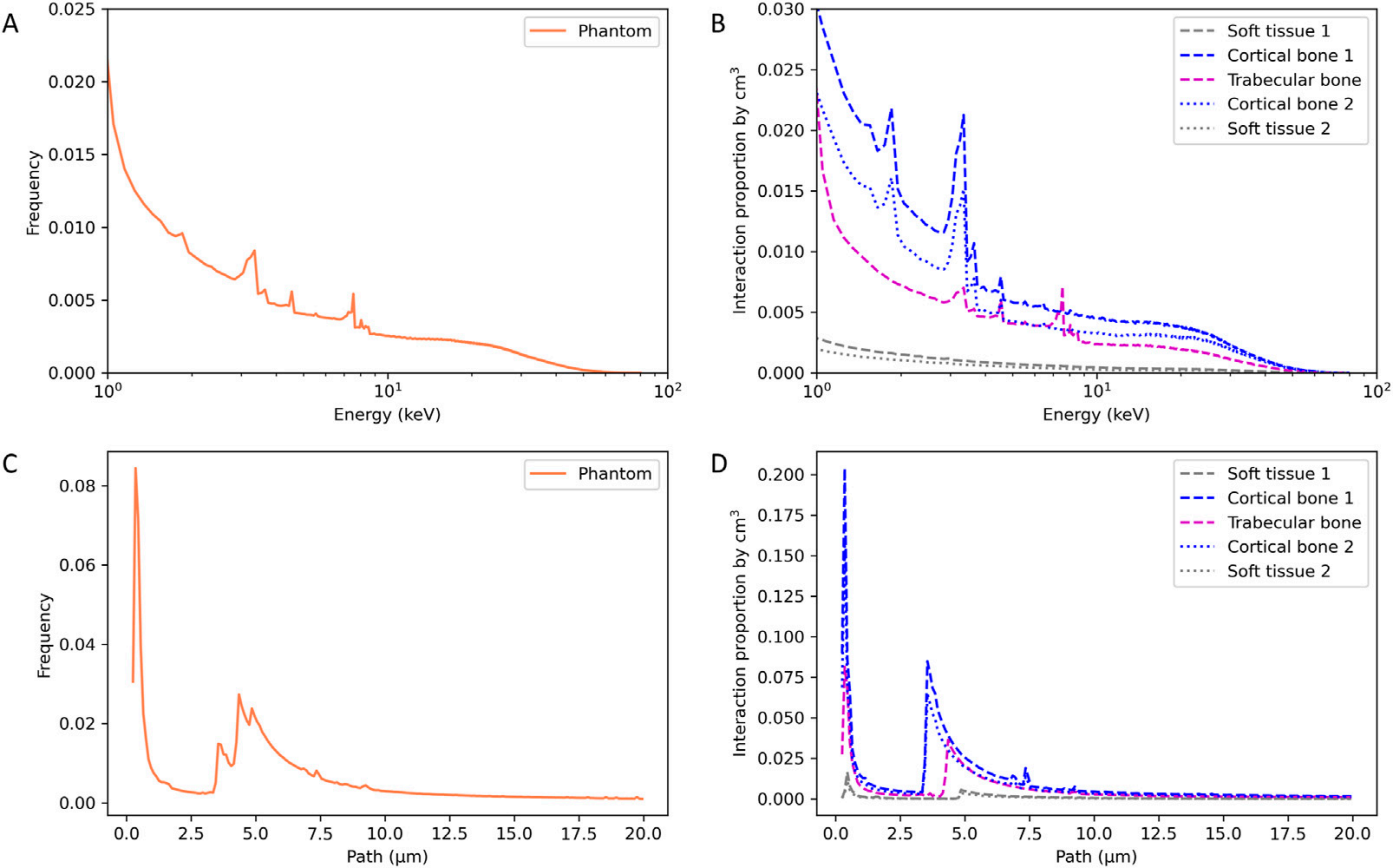
Energy spectrum **(A,B)** and path **(C,D)** of secondary electrons in the heterogenous simplified phantom.

The characteristics of the secondary electrons were studied in the simplified heterogenous phantom, allowing for extensive statistical analysis in the different tissues. Figure 5 reports the secondary electron energy spectrum and paths in the total phantom (Figures 5A, C) and for each tissue (Figures 5B, D). For this study, the interaction frequency was normalized by the total number of interactions in the total phantom (A and C) and by the volume of each tissue (B and D).

The secondary electron energy spectrum in the total phantom shows the decrease of the electron’s frequency with increasing energy, as expected, and specific energy peaks. By observing the decomposition of the energy spectrum according to the type of tissue, we can identify the specificities for each material. Cortical bone shows energy peaks at 1.85, 3.35, 3.65 and 4.55 keV, trabecular bone shows energy peaks at 3.35, 4.55, 7.55 and 8.05 keV and no peaks for the soft tissue. Electrons having an energy of 1.85 and 3.65 are specific to cortical bone and electrons having an energy of 7.55 and 8.05 keV are specific to trabecular bone.

Concerning the path of secondary electrons, specific paths at 3.55, 4.35 and 4.85 µm have been identified in cortical bone, trabecular bone, and soft tissue, respectively. This specific path increases as the density of the considered tissue decreases.

**FIGURE 6:**
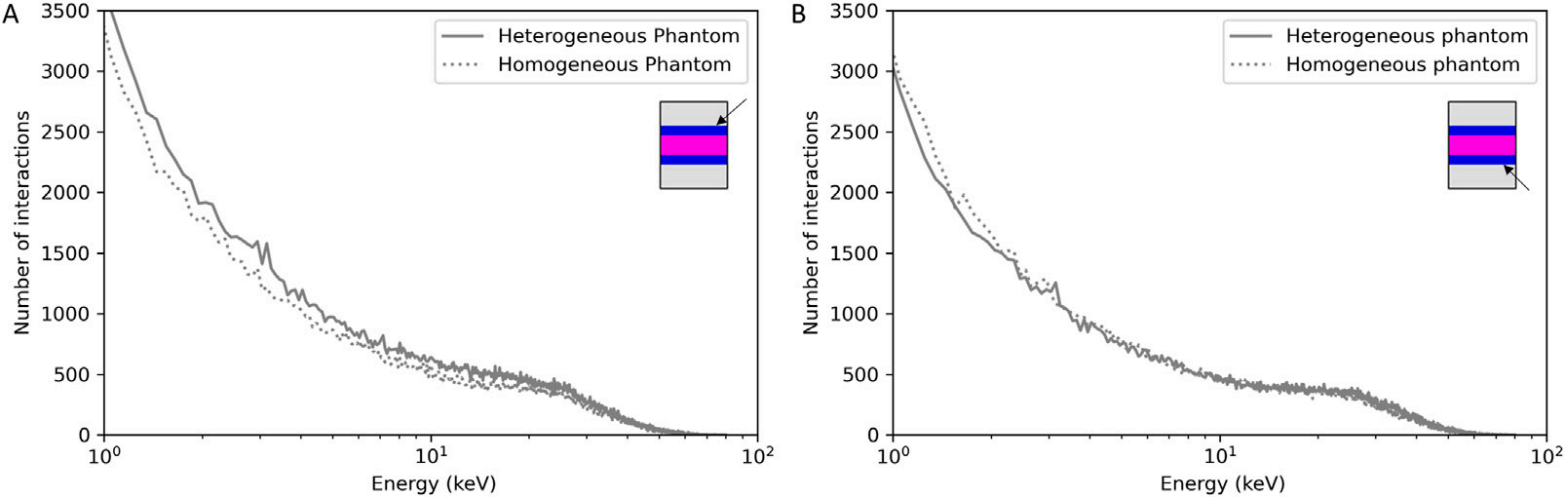
Frequency of secondary electrons as a function of their energies in the 100 µm soft tissue slice before **(A)** or after **(B)** cortical bone compared to a homogeneous soft tissue phantom at the same position.

Figure 4A showed a slightly higher dose deposition in the 100 µm tissue slices in contact with the cortical bone. Following this observation, we evaluated if we had a modification of the energy or of the path of secondary electrons. Figure 6 reports the energy spectrum of secondary electrons in the soft tissue slices in contact with the cortical bone compared to a phantom composed only of soft tissue at the same position.

In the soft tissue slice before cortical bone 1 (Figure 6A), we can identify secondary electrons having the specific energies of the cortical bone (3.35 and 3.65 keV). Secondary electrons coming from cortical bone one interact in the soft tissue, which is not the case for the soft tissue slice after cortical bone 2 (Figure 6B). No difference was observed for the secondary electron paths (data not shown).

## 4 Discussion

This work was initiated in the framework of the development of a new mouse preclinical model on the SARRP platform to mimic radiological burn under interventional radiology conditions. Indeed, interventional radiology has become an essential tool for the diagnosis and treatment of various pathologies. This technique relies on the use of low-energy X-rays imaging and, although largely mastered, high local dose deposition can be delivered to the patient, leading to overexposure and the appearance of deterministic effects ranging from simple erythema to tissue and/or bone radionecrosis in the most severe cases (Coeytaux et al., 2015; Jaschke et al., 2017). The lack of knowledge about the biological consequences at low energy, due to the heterogeneity of dose deposition, makes the prognosis very uncertain for the different tissues and especially for bone. Characterizing the biological effects of this type of exposure is essential to improve patient management and risk prediction. To this end, it was necessary to develop an adapted preclinical model to mimic interventional radiology overexposure.

Many preclinical models have already been proposed in the literature in both rats and mice to improve knowledge and to better understand the radiopathological specificities of ionizing radiation on skin, muscle and/or bone tissues using different qualities of radiation (Lerouxel et al., 2009; Jang et al., 2016; Rottensteiner-Brandl et al., 2017; Miller et al., 2019; Zhai et al., 2019; Dos Santos et al., 2021b). With the democratization of the use of conventional X-rays cabinets or dedicated image-guided platforms, these types of facilities are more and more widely used for radiobiological studies (Brown et al., 2022) and are particularly well-suited for our study as they involve low-energy X-rays. Although several models exist, few of them have been implemented in the specific range of energies used in interventional radiology (70–120 kV) or used to perform a complete characterization of the dose deposition in different tissues. Moreover, it is sometimes difficult to know exactly how the dosimetry measurements were performed and what quantity was measured (Draeger et al., 2020). Today, the importance of dosimetry for radiobiological studies no longer requires demonstration and several studies have highlighted the importance of properly defining the irradiation conditions and how dosimetry measurements were performed. Exact knowledge of the delivered doses is crucial, especially when low X-rays energies are involved where the photoelectric effect is predominant, leading to a strong heterogeneity in dose deposition depending on the density and composition of the tissue traversed. Monte Carlo simulations contribute considerably to the understanding of how the dose is deposited for these low energies, according to tissue type. Several studies over 100 kV have already shown the dose enhancement on bone tissues (Chow, 2010; Chow and Jiang, 2012; Poirier et al., 2020). Combining experimental dosimetry measurements with this type of Monte Carlo simulation is a considerable asset for the implementation of new preclinical models for radiobiological studies.

In this study, we proposed a complete dosimetric characterization of this new preclinical model using Monte Carlo simulations and experimental measurements by EPR spectroscopy. A new irradiation configuration was implemented on the Small Animal Radiation Platform (SARRP) using a voltage of 80 kV and an additional filtration of 0.15 mm Cu to achieve an energy spectrum in the same range as those used in interventional radiology (70–120 kV). Irradiation setup was optimized to be able to perform localized irradiation of the mouse hind leg, the source distance detector was reduced, and a specific brass collimator was designed (Figure 1).

### 4.1 Experimental bone dose estimation

Experimental dosimetry measurements by EPR spectroscopy were carried out to estimate the bone dose. Although this method is invasive and requires the use of specific animals as the bones have to be extracted, it is a considerable asset for dose determination in biological tissue, allowing evaluation of inter-mouse and inter-sample variability and the establishment of new preclinical models. 15 mice were exposed to a single dose of 30 Gy in air kerma and 30 tibia bones were harvested for dose estimation. Considering all the samples, a bone dose of 194.0 ± 27.0 Gy was measured and an experimental conversion factor between the bone and the air dose of 6.5 ± 0.9 was defined, highlighting the heterogeneity of the dose deposit depending on the material considered. An inter-sample variability of 14.5% and 12.6% for right and left tibia bones, respectively, was obtained. Using the dose estimation in right and left tibia bones for a single mouse, we have also highlighted the intra-mouse variability ranging from 0.4% to 21.9%. The uncertainties obtained experimentally by EPR spectroscopy for the bone dose remain higher compared to uncertainties with physical reference detector (less than 5% for ionization chamber at k = 2) but we have to keep in mind that measurements are performed in biological samples. It is well known that parameters such as age, sex or strains impact a lot of physiological parameters in mice, including the bone composition, density, or mineral contents (Akhter et al., 2000). Some studies have performed measurements on the bone mineral content and mineralization showing that for C57BL6J mice, used in this work, variation of up to 9% in bone mineral content and bone density may be present (Akhter et al., 2000; Somerville et al., 2004). These variations in bone composition and density of the bone sample strongly impact the dose deposition measured on our tibia bones. Moreover, experimental (EPR) or calculated studies on the effects of different qualities of X-rays have highlighted the dose enhancement on bone tissues at low energy and also the importance of taking into account the bone composition in the calculation of the absorbed dose (Copeland et al., 1993; Johnson et al., 2011).

### 4.2 Dosimetric characterization by monte carlo simulations

The dose deposition in the different tissues (bone, soft tissue) and to conduct an analysis of the secondary electrons created to better understand how the dose deposition is done and the specificities of the electrons involved as there are the main responsible of the dose deposition, Monte Carlo simulations were performed. First, the whole geometry of the irradiation facility was introduced on the simulations to create a source model in order to save computation time. The analysis of the source model was performed by comparing the X-ray energy spectrum, the HVL and the momentums to the real source and experimental or SpeckCalc software data (Poludniowski et al., 2009). For the energy spectrum and the HVL, a fairly good agreement was found between the simulated data and the experimental data or Spekcal software outputs. The analysis of the photon momentums allowed to reduce the source divergence as much as possible without affecting the irradiation field to save computation time. Indeed, the dose profile of the homemade brass collimator was characterized by using EBT3 radiochromic films and compared to simulated data (Figure 3). Good agreement was obtained, compliant with IAEA criteria, except for the outside beam edge criterion. This discrepancy in the profile tails may be due to the geometry introduced in geant4 or to the size/shape of the virtual source defined but as the phantoms used to evaluate the dose deposition were smaller than the irradiation field (1 cm margin) and placed in the full center, we chose not to modify the source parameters or the implemented collimator. The dose deposition in the different phantoms highlighted the heterogeneity of the dose deposition for these low X-ray energies (Figure 4). Using the simplified phantoms, the attenuation was quantified and is dependent on the density and thickness of the tissue penetrated, in agreement with the results of literature where calculations with 105 and 225 kV photon beams were performed in different materials (Chow and Jiang, 2012). We also compared the results of our Monte Carlo simulations with our experimental measurements by comparing, in particular, the bone to air conversion factor. A mean bone to air conversion factor of 6.0 ± 0.5 in the heterogeneous phantom, 6.2 ± 1.0 for cortical bone in the voxelized phantom and 6.5 ± 0.9 for experimental data. A fairly good agreement was obtained even if a unique trabecular or cortical bone density and composition was used for Monte Carlo simulations, validating our calculated results. Indeed, in the kilovoltage range, the importance of the density and the composition of the material is crucial for dose estimation. As reported by Zhou et al. (Zhou et al., 2009), calcium and phosphorus are strongly correlated to bone density and impact the mass attenuation coefficient. Moreover, even if the mass attenuation coefficient does not vary more than 10% for photon energy of more than 200 keV, this value is several times higher for lower photon energy. Thus, a variation in the quantity of these two elements can strongly impact the bone composition (Zhou et al., 2009). Verhaegen and Devic also reported that errors in dose estimation of up to 40% may occur for 250 kV X-rays (Verhaegen and Devic, 2005). Performing MC simulations with different bone densities would be very time-consuming, but we performed simplified calculations in homogeneous phantoms with a thickness of 7 mm (mouse leg) where we varied the amount of calcium or the density of the bone. These results show that the attenuation can vary from 10% (lower bone density) to 80% (only calcium), and the dose deposition for different bone compositions can vary from 3 to 20% depending on the depth (Figure 2B). These observations support the fact that experimental measurements are undeniable assets for the dosimetric characterization of new preclinical models and that they allow biological differences to be taken into consideration.

### 4.3 Specificities of secondary electrons

Simulations in the heterogeneous phantom also allowed us to study the specificities of secondary electrons, which are the main cause of dose deposition. As reported in Figure 5, the proportion, the path, and the energies of the secondary electrons created are dependent on the material. Comparing trabecular and cortical bone, we can observe that some characteristic rays are specific to the density and composition of the bone, supporting the fact that an accurate estimation of the bone composition is required for the most efficient dose estimation by Monte Carlo simulations. Lastly, we evaluated the specificities of the soft tissue slice in contact with the cortical bone. In the 100 µm soft tissue slice before the cortical bone we have an increase of 10% in dose deposition due to the backscatter electrons (Figure 4B). In the soft tissues after the cortical bone, we have a decrease of dose deposition of 20% due to the attenuation, except for the first 100 µm soft tissue slice (in contact with the cortical bone) where the decrease is only about 10% due to the contribution of the forward electrons. These results are in agreement with the publication of Das and Chopra where dose enhancement and the contribution of secondary electrons due to the presence of high Z material was studied (Das and Chopra, 1995; Das, 1997). Thus, as the secondary electrons created on bone contribute to the dose deposition in the first micrometer of soft tissue in contact, in the event of over exposure in interventional radiology, more damage can be found in these tissues and they should be given specific consideration.

## 5 Conclusion

This work proposes a new preclinical model to perform overexposure under interventional radiology conditions. A complete Monte Carlo characterization of the model has been performed in terms of dose deposition and secondary electron characterization showing the influence of the material density and composition when low-energy X-rays are involved. Moreover, with the proposed approach, it is possible to determine the dose deposited on mouse bones experimentally by EPR spectroscopy when irradiated using IR-like conditions on the SARRP platform and validate the Monte Carlo calculation results. Taken together, these results strongly support the argument that experimental dose estimation is essential for the implementation of new radiobiological models and justify performing a dose estimation by EPR spectroscopy, especially at low-energy X-rays where the density and composition of the material considered play a major role in dose deposition.

## Data Availability

The original contributions presented in the study are included in the article/supplementary material, further inquiries can be directed to the corresponding author.
